# Exploring the Potential Antidepressant Mechanisms of TNFα Antagonists

**DOI:** 10.3389/fnins.2019.00098

**Published:** 2019-02-11

**Authors:** Kyle J. Brymer, Raquel Romay-Tallon, Josh Allen, Hector J. Caruncho, Lisa E. Kalynchuk

**Affiliations:** ^1^Department of Psychology, University of Saskatchewan, Saskatoon, SK, Canada; ^2^Division of Medical Sciences, University of Victoria, Victoria, BC, Canada

**Keywords:** depression, inflammation, cytokines, stress, TNF-α, rat, antidepressant, hippocampus

## Abstract

Human and animal studies suggest an intriguing relationship between the immune system and the development of depression. Some peripherally produced cytokines, such as TNF-α, can cross the blood brain barrier and result in activation of brain microglia which produces additional TNF-α and fosters a cascade of events including decreases in markers of synaptic plasticity and increases in neurodegenerative events. This is exemplified by preclinical studies, which show that peripheral administration of pro-inflammatory cytokines can elicit depression-like behavior. Importantly, this depression-like behavior can be ameliorated by anti-cytokine therapies. Work in our laboratory suggests that TNF-α is particularly important for the development of a depressive phenotype and that TNF-α antagonists might have promise as novel antidepressant drugs. Future research should examine rates of inflammation at baseline in depressed patients and whether anti-inflammatory agents could be included as part of the treatment regimen for depressive disorders.

## Depression

Depression remains the most common psychiatric disorder, affecting approximately 350 million people worldwide. Accordingly, depression now ranks as the top cause of global disability in terms of years lost due to disability. The defining characteristics of depression include anhedonia, a loss of interest in pleasurable activities, and lowered mood (Nestler et al., 2002). Depression is further characterized by alterations in cognition, weight, sleep, irritability, thoughts of suicide, and decreased sexual function/interest (Nemeroff, 1998). Medications that target the monoaminergic system provide the primary course of treatment for depressed patients. These medications include monoamine oxidase inhibitors (MOAI’s), tricyclic antidepressants (TCAs), and selective serotonin reuptake inhibitors (SSRI’s). Despite their widespread use, currently available antidepressants are frequently ineffective, with the percentage of patients experiencing remission as low as 45% (Thase et al., 2001). This suggests that factors other than disruptions in monoamines are at play in the development of depressive symptoms. Indeed, recent evidence has implicated astroglial pathology (Wang et al., 2017), mitochondrial dysfunction (Allen et al., 2018), and deficient hippocampal neurogenesis and neuronal maturation (Bessa et al., 2009; Eisch and Petrik, 2012; Lussier et al., 2013; Mateus-Pinheiro et al., 2013) as potential causal factors in depression. In addition, it has long been thought that neuroinflammation plays a key role in the etiology of depression (Brites and Fernandes, 2015; Furtado and Katzman, 2015). In this short review, we focus on new data suggesting that elevations in the inflammatory cytokine TNF-α might instigate depressive symptoms and that therapies aimed at reducing TNF-α levels hold therapeutic promise.

## The Role of Inflammation in Depression

**FIGURE 1:**
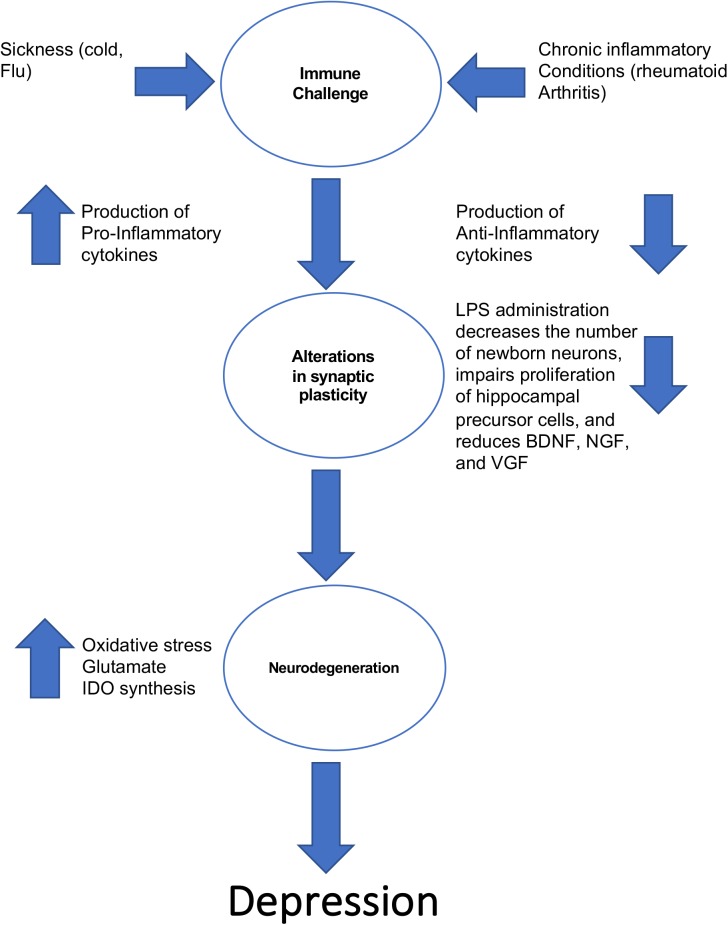
The ways in which inflammation can lead to the development of depressive-like behavior.

The notion that inflammation could be a contributing factor in the pathogenesis of depression is not a new one. Work in the 1980s’s first revealed that some patients with heightened immune activity (e.g., a patient with an intense cold) displayed the hallmark features of depression (i.e., lethargy, depressed mood, anhedonia) (Miller and Raison, 2016). This in turn led to the cytokine sickness hypothesis of depression, which posits that sustained increases in circulating levels of pro-inflammatory cytokines can produce depressive symptoms (Dantzer, 2009). This hypothesis is summarized in Figure 1. Interestingly, high circulating levels of the pro-inflammatory cytokine IL-6 are strongly associated with feelings of guilt and suicidal ideation (Alesci et al., 2005; O’Donovan et al., 2013). If the cytokine sickness hypothesis of depression is correct, then one would expect that disorders characterized by high circulating levels of cytokines would share a high comorbidity with depression, and this turns out to be the case. For example, rheumatoid arthritis, which is a disorder in which the immune system targets bodily tissues and instigates widespread inflammation, shares a 13–42% comorbidity rate with depression (Margaretten et al., 2011). Furthermore, cancer patients treated with cytokines experience a significant reduction in plasma tryptophan levels that coincides with depression (Capuron et al., 2002). This tryptophan reduction reduces the bioavailability of serotonin, which is a known risk factor for the development of depression according to the monoamine hypothesis. It was later discovered that the culprit for this decrease in plasma tryptophan levels in cancer patients receiving immunotherapy is indoleamine 2,3-dioxygenase (IDO). Activation of IDO decreases tryptophan bioavailability, creating a net decrease in monoamines (Dantzer, 2009). Immuno-activation in healthy control subjects creates depressive-like behavior and impairments in cognition (Reichenberg et al., 2001), and serum concentrations of interleukin-6 at 9 years of age is positively correlated with depressive symptomology at 18 years of age (Khandaker et al., 2014). Finally, high circulating levels of cytokines are associated with treatment-resistant depression (Carvalho et al., 2013).

Stress is also known to transiently elevate the expression of pro-inflammatory cytokines. Unsurprisingly, pro-inflammatory cytokines are efficient activators of the hypothalamic-pituitary-adrenal (HPA) axis (Kenis and Maes, 2002), which often becomes dysregulated in depression. However, as not all depressed patients display a dysregulated HPA axis, it is tempting to speculate that elevated levels of pro-inflammatory cytokines occur in a subset of depression patients, possibly those patients who develop symptoms after a period of major life stressors leading to HPA dysfunction.

## Inflammation and Cytokines: the Roles of TNF-α

Cytokines are increasingly being recognized as having a contributive role in the development of depressive symptoms. Treatment with proinflammatory cytokines, including IL-1, IL-6, or TNF-α, or lipopolysaccharide (LPS), induces sickness behavior and corresponding depression-like behavior on the forced swim test (Dantzer, 2004; Dunn and Swiergiel, 2005; Henry et al., 2008). Yang et al. (2015) showed that chronic mild stress (CMS) resulted in an increased expression of proinflammatory cytokines, particularly IL-1, IL-6, and TNF-α, and lower immunoreactivity of myelin protein and decreased numbers of oligodendrocytes in the prefrontal cortex that coincided with the development of depression-like behavior. Maldonado-Bouchard et al. (2015) showed that the stress associated with lesioning the spinal cord in rodents resulted in increased levels of hippocampal cytokines and increased depression and anxiety-like behaviors. This suggests that the inflammation and increased cytokine release *per se* produced by a spinal cord injury can lead to the development of depression-like behaviors. Dunn and Swiergiel (2005) demonstrated that mice treated with IL-1 spent significantly more time immobile on both the forced swim test and tail suspension test, which are two classic rodent indices of depression-like behavior. Mice that lack certain cytokines or cytokine receptors do not display stress-induced depression-like behavior (Chourbaji et al., 2006), which suggests that lower levels of cytokines confer a protective effect on the development of depression-like behavior. The idea that low levels of cytokines could protect against the development of depression-like behavior is an interesting one and one that will be explored in greater detail in subsequent sections of this review.

Although the release of pro-inflammatory cytokines can contribute to the development of depression-like behavior, TNF-α in particular is receiving considerable attention due to its prominent roles in promoting inflammation and its dampening effects on synaptic plasticity (Khairova et al., 2009; Pribiag and Stellwagen, 2014; Lewitus et al., 2016). It is important to differentiate between TNF-α in the periphery and TNF-α in the brain. Recent findings suggest TNF-α is produced peripherally by leukocytes, lymphoid cells, mast cells, endothelial cells, and adipose tissue and is involved in functions of host defense including the stimulation of protective granuloma formation incurred during mycobacterial infections and the promotion of liver and spleen function (Kruglov et al., 2008). However, when TNF-α signaling is not tightly controlled, dysregulation of peripheral TNF-α signaling can contribute to the development of inflammatory and autoimmune disorders including septic shock and rheumatoid arthritis (Kruglov et al., 2008).

TNF-α is a protein that is initially released as a soluble cytokine (sTNF-α) after being enzymatically cleaved by its cell surface bound precursor (tmTNF-α) by TNF-α converting enzyme (TACE) (Bortolato et al., 2015) and is therefore expressed as a transmembrane protein. TNF-α binds to one of two receptors: TNF receptor 1 (TNFR1) and TNF receptor 2 (TNFR2). TNFR1 is activated by soluble and transmembrane TNF-α, and promotes inflammation and tissue degeneration (Kalliolias and Ivashkiv, 2016). TNFR2’s expression is restricted to neurons, endothelial cells, and immune cells, and is involved in mediating cell survival and tissue regeneration (Kalliolias and Ivashkiv, 2016). The sTNF-α possesses a higher affinity for binding with TNFR1. When TNF-α binds to TNFRs, complex 1 is assembled at the plasma membrane and includes the TNF-α associated death domain protein (TRADD) among other complexes, resulting in the creation of a scaffolding ubiquitin network (Kalliolias and Ivashkiv, 2016). This scaffolding ubiquitin creates the recruitment and activation of two signaling complexes: transforming growth factor (TGF) -β activated kinase 1 (TAK1) complex and the inhibitor of kB (Ikkβ) kinase complex (Kalliolias and Ivashkiv, 2016).

One of the main roles of TNF-α is in maintaining inflammation during times of proinflammatory conditions. During proinflammatory events, TNF-α production is induced by other cytokines (e.g., IL-1) and microglia. Once released, TNF-α stimulates the production of other proinflammatory cytokines, including IL-1 and 6, and it increases the production of reactive oxygen intermediates, including nitric oxide (Bortolato et al., 2015). It is easy to conceptualize this process as a positive feedback loop, whereby an initial stressful or inflammatory event triggers the release of TNF- α, which in turn triggers the release of other pro-inflammatory cytokines, creating a state of prolonged inflammation. This helps explain, why autoimmune diseases are among the hardest disorders to treat. Perhaps it is not surprising that increased inflammation as a result of sustained TNF-α production and release results in altered glutamatergic signaling and excitotoxicity. Mechanistically, TNF-α upregulates glutaminase (the enzyme responsible for the conversion of glutamate from glutamine) expression, resulting in the transportation of glutaminase from the mitochondria into the extracellular space. This in turn leads to elevated concentrations of glutamate both intracellularly and extracellularly, eventually causing cell death through excitotoxicity (Ye et al., 2013). This fits in line with the reported elevations of plasma glutamate levels seen in depressed populations (Inoshita et al., 2018). Interestingly, proinflammatory cytokines (TNF-α) trigger the release of kidney type glutaminase (KGA) from mitochondria, which then travels to the cytosolic compartment of neurons (Ye et al., 2013), increasing glutamate content. This is of interest as we have recently published a report outlining a link between mitochondrial function and depression (see Allen et al., 2018).

## TNF-α and Depression: Animal Models and Clinical Studies

Preclinical studies corroborate the role of TNF-α in depression-like behavior. Peripheral administration of TNF-α can produce anhedonic behavior in rodents (van Heesch et al., 2013). Likewise, deletion of TNFR1 or TNFR2 creates an antidepressant phenotype on measures of depressive-like behavior (Simen et al., 2006). Yamada et al. (2000) showed that TNF-α knockout mice display a mild antidepressant phenotype. Along these same lines, administration of the TNF-α inhibitor infliximab during chronic mild stress significantly decreased immobility time in the forced swim test, increased sucrose consumption during the sucrose preference test, and decreased anxiety-like behavior in the elevated plus maze (Karson et al., 2013).

Given that cytokine treatment produces depression-like behavior and reducing cytokine levels alleviates this, the question that arises is whether or not antidepressants can influence inflammation. Interestingly, SSRIs and tricyclic antidepressants are known to reduce levels of TNF-α and other proinflammatory cytokines and increase anti-inflammatory cytokines, including IL-10 (Song et al., 1994). Two potential explanations for how antidepressant drugs might reduce pro-inflammatory cytokine levels have been offered. The first explanation posits that higher levels of activation of serotonergic receptors located on immune cells dynamically regulate the production of pro-and anti-inflammatory cytokines, and this could be influenced by a higher availability of serotonin upon antidepressant treatment, although the pro- or anti- inflammatory effects of peripheral serotonin is still under debate (recently reviewed in Herr et al., 2017; see also Kenis and Maes, 2002). The second explanation sees the increased production of cyclic adenosine monophosphate (cAMP) by antidepressants as a mechanism by which antidepressants might reduce cytokine levels. Specifically, cAMP activates protein kinase A (PKA), increasing the production of cAMP responsive element binding protein (CREB), both of which act to decrease pro-inflammatory cytokine production (Kenis and Maes, 2002).

Considerable attention has been dedicated to the antidepressant potential of ketamine. Ketamine rapidly reverses depression-like behavior in both clinical and preclinical subjects, in a timeframe of hours (Yoshi and Constantine-Paton, 2010; Cusin et al., 2012). An important question asked within the literature is whether ketamine is altering neuroinflammation? Although this question is relatively recent, it does appear to be the case that at least a component of the antidepressant properties of ketamine involve reductions in neuroinflammation, in particular TNF-α. For example, Wang et al. (2015) have shown that in chronically-stressed rats, the rapid antidepressant effects of ketamine are accompanied by a reduction of hippocampal TNF-α levels. Moreover, a reduction in depressive symptoms 40 min post-ketamine infusion in depressed patients has been shown to be correlated with reductions in serum TNF-α levels (Chen et al., 2018). These findings suggest that rapid changes in neuroinflammation, in particular TNF-α, are perhaps one of the mechanisms underlying the antidepressant actions of ketamine.

In terms of clinical studies, a large body of evidence supports the role of TNF-α in depression. Endotoxin administration in control subjects produces an increase in TNF-α in addition to depressed mood and cognitive impairment (Della and Hannestad, 2010). Microarray mRNA studies demonstrate increased expression of tmTNF-α in the prefrontal cortex (PFC) of suicide victims (Pandey et al., 2012). In fact, higher suicidal ideation itself is associated with an increased cytokine profile, including elevated TNF-α (O’Donovan et al., 2013), and high circulating levels of TNF-α are found in peripheral tissues of suicide victims (Lindqvist et al., 2009). Several lines of evidence support the efficacy of TNF-α inhibitors in the treatment of depression. Patients with rheumatoid arthritis and plaque psoriasis taking prescribed etanercept, which is a TNF-α antagonist, reported significant reductions in depressive symptoms (Gelfand et al., 2008; Kekow et al., 2011). Similarly, patients with Crohns Disease receiving infusions of infliximab experienced significant reductions in depressive symptoms and this decrease was associated with corresponding reductions in proinflammatory cytokines (Guloksuz et al., 2013). Finally, psoriasis patients with comorbid psychiatric conditions report improvement in mood and overall well-being when taking infliximab (Bassukas et al., 2008). Interestingly, inflammation itself is associated with anhedonia, and one of the first symptoms to be alleviated in depressed patients receiving anti-inflammatory compounds is anhedonia (Felger et al., 2016).

Some interesting extensions of these clinical studies have been observed in patients with treatment-resistant depression. Raison et al. (2013) reported that patients with treatment -resistant depression with a high baseline level of inflammation as indicated by elevated high sensitivity C-reactive protein expression responded favorably to infusions of the TNF-α inhibitor infliximab. However, in patients with a low baseline level of high sensitivity C-reactive protein infliximab was not more effective than placebo. This pattern of results suggests that subsets of treatment-resistant patients experience high levels of inflammation, and therapies aimed at reducing inflammation might be particularly effective in these patients. However, other patients may have a different physiological profile such that factors other than inflammation are at play. This conclusion is consistent with the observation that two depressed patients can present with a different cluster of symptoms. For example, patient A could present with agitation, weight loss, an inability to sleep, and lowered mood, whereas patient B presents with weight gain, excess sleep, psychomotor retardation, and anhedonia. This raises the question (asked elsewhere; see Nestler and Hyman, 2010) of whether depression is actually part of a constellation of different disorders. It is tempting to suggest that depressed patients with inflammation might represent a subset of depressed patients who require a different course of treatment compared to their non-inflamed counterparts. Kappelmann et al. (2016) conducted a meta-analysis of the effectiveness of anti-cytokine treatments in depression. They found that across seven double-blind clinical trials involving 1309 subjects, anti-cytokine treatment was generally more efficacious than treatment with placebo. Of interest is the fact that several research groups have reported that currently available non-cytokine antidepressant medications are not more effective than placebo, at least in the treatment of non-severe depression (Garland, 2004; Kirsch et al., 2008; Fournier et al., 2010). Therefore, treatments that target inflammation might represent a more viable approach to the treatment of depression, as the monoamine hypothesis of depression largely does not align with what is currently known about the biological causes of depression. Of the anti-cytokine treatments analyzed, Kappelmann et al. (2016) reported that anti-TNF-α drugs were the most commonly used option. The picture that emerges is that depression is associated with elevations in TNF-α, and treatments aimed at reducing circulating TNF-α produce significant normalization of depressive symptoms. In the next section, we elaborate on some putative mechanisms underlying the antidepressant effect of TNF-α antagonists.

## Targeting TNF-α in Depression

Peripheral injection of TNF-α antagonists (i.e., etanercept) causes a functional decrease in peripheral TNF-α with only an indirect effect on central TNF-α expression, as drugs like etanercept cannot cross the blood brain barrier (Boado et al., 2010). Therefore, it has been assumed that drugs like etanercept are only able to indirectly reduce central inflammation as a consequence of reduced peripheral TNF-α activity (Kerfoot et al., 2006). However, TNF-α *per se* can cross the blood brain barrier by a receptor mediated mechanism (Pan and Kastin, 2001, 2002; Pan et al., 2006), and when this occurs, it instigates an increase of both TNF-α protein and mRNA by stimulating central expression of TNF-α by microglial cells (Qin et al., 2007; McCoy and Tansey, 2008). Peripheral TNF-α also can stimulate secretion of TNF-α from circumventricular organs and choroid plexus, and TNF-α secreted by these organs can then induce the activation of microglia and a subsequent increase in TNF-α secretion by microglial cells (Qin et al., 2007; McCoy and Tansey, 2008). As stated, etanercept does not cross the blood brain barrier but it binds peripheral TNF-α, and in doing so, it reduces the effect of peripheral TNF-α in promoting the activation of microglia, which results in decreased secretion of central TNF-α.

One should therefore expect that etanercept injections would prevent some of the central effects of protracted release of central TNF-α, such as its effects on hippocampal activity and neurogenesis (see McCoy and Tansey, 2008; and Bortolato et al., 2015). In line with this observation, we have recently shown that semi-weekly peripheral injections of etanercept (0.8 mg/kg) can normalize the depression-like behavior produced by 21 days of exogenous corticosterone injections in rats (Brymer et al., 2018). Etanercept can also restore performance on object-location and object-in-place recognition memory tests of hippocampal functioning. Moreover, etanercept restores the number and complexity of dentate subgranular/granular neurons expressing doublecortin, which is a marker of immature newborn neurons, and perhaps this action of etanercept may be underlying its antidepressant effects (Brymer et al., 2018). It should in any case be noted that the role of adult hippocampal neurogenesis in depression remains a contentious issue. Antidepressant effects can be achieved without increases in neurogenesis, and ablation of neurogenesis is not sufficient to create a depression-like phenotype (Hanson et al., 2011). Recently, Sorrels et al. (2018) found that human hippocampal neurogenesis sharply drops from childhood to near undetectable levels in adulthood, boldly suggesting that hippocampal neurogenesis does not occur past childhood. However, counter to this report, Boldrini et al. (2018) report that human hippocampal neurogenesis persists throughout the lifespan, even into the 70th year of life. Moreover, recent reports have found reductions in hippocampal neurogenesis in post-mortem tissue from patients with depression (Boldrini et al., 2012). In an elegant study, Hill et al. (2015) showed that increasing hippocampal neurogenesis through transgenic methods alone is sufficient to create an antidepressant phenotype. While the debate seems far from over, the picture that emerges is that neurogenesis is associated with depression, however, the degree of causality in human populations is still unknown.

Another intriguing hypothesis about the mechanism by which etanercept might enhance hippocampal neurogenesis comes from a separate set of studies we conducted that focus on the extracellular matrix protein reelin. Reelin has been extensively studied for its role in guiding cell migration during development, but in the adult brain it is involved in the promotion of synaptic plasticity. Reelin binds to two receptors, the very-low-density lipoprotein receptor (VLDR) and apolipoprotein receptor 2 (ApoER2). Activation of these receptors by reelin ultimately excites downstream targets including mTOR and P13K (Jossin and Goffinet, 2007). Importantly, inactivation of either PI3K or mTOR has been shown to reduce dendritic complexity in neuronal cultures (Jaworski et al., 2005). On a more direct level, reelin overexpression accelerates dendritic growth within adult-generated neurons, and inactivation of the reelin signaling pathway impairs adult hippocampal neurogenesis (Teixeira et al., 2012). In addition to our findings that peripheral etanercept injections can normalize neurogenesis after a period of corticosterone administration, we also showed that etanercept rescues reelin expression in GABAergic interneurons located in the proliferative subgranular zone of the dentate gyrus (Brymer et al., 2018). We have previously hypothesized about the important role that reelin could play in the neurobiology of depression (Caruncho et al., 2016), as revealed by our observations that depression-like behavior is associated with a significant decrease in the number of reelin+ cells in the subgranular zone (Lussier et al., 2009; Fenton et al., 2015). We found that the timecourse for the emergence of depression-like behavior after corticosterone administration parallels the timecourse for dampened neurogenesis and the loss of reelin-positive cells (Lussier et al., 2013). We also found that heterozygous reeler mice, with 50% normal levels of reelin, were more susceptible to the depressogenic effects of corticosterone than wild type mice (Lussier et al., 2011). Taking all these observations into account, we believe that the antidepressant effects of etanercept in rats treated with corticosterone could occur through a normalization of hippocampal reelin expression. This is an important area for future studies as it could help explain the mechanism by which TNF-α antagonists exert their antidepressant effects.

It is therefore worth considering how etanercept might interact with the reelin signaling system in the dentate gyrus. Very little direct research has been done on this topic to date, but there could be a link through neuronal nitric oxide. TNF-α promotes the expression of nitric oxide synthase, and there are reports that some neuronal subtypes might reflect nitric oxide-mediated oxidative damage in response to increased levels of TNF-α (see as example Thomas et al., 2005). There is also evidence that the nitric oxide system has multiple effects in modulating adult neurogenesis (reviewed in Carreira et al., 2013). Overproduction of nitric oxide and accumulation of nitric oxide metabolites has been linked to mitochondrial dysfunction and oxidative stress in depression (recently reviewed by Allen et al., 2018). Interestingly, we have found that heterozygous reeler mice, which as mentioned above are highly susceptible to the depressogenic effects of repeated corticosterone (Lussier et al., 2011), show a decrease in the number of neurons co-expressing reelin and neuronal nitric oxide synthase (nNOS) specifically in the proliferative subgranular zone (Romay-Tallon et al., 2010). We also know that repeated corticosterone has differential effects on the co-expression of reelin and nNOS in wild type and heterozygous reeler mice (Romay-Tallon et al., 2015). We have discussed these data as an indication that nitric oxide-mitochondria-mediated excitotoxic events in reelin expressing neurons in the subgranular zone may instigate a decrease in reelin secretion by these neurons, resulting in deficits in dendritic maturation within newborn granule cells and dampened hippocampal plasticity that may be a key event in the pathophysiology of depression (see Caruncho et al., 2016; Allen et al., 2018). One could therefore surmise that the effects of etanercept in antagonizing the actions of TNF-α could result in a reversal of its effects in activating the NO system, and a subsequent neuroprotective action on reelin expressing neurons in the dentate subgranular zone. Additional research is necessary to properly assess these hypotheses, but they could open the door to the identification of novel targets for new antidepressant drug development.

One should also consider that there seems to be considerable cross-talk between ApoE and TNF-α in that low levels of ApoE result in increased cytokine production, in particular TNF- α and that in a similar vein, release of proinflammatory cytokines (TNF-α) downregulates ApoE production (Zhang et al., 2011). It is therefore likely that under conditions of stress, such as during administration of exogenous corticosterone, peripheral production and release of TNF-α is enhanced, which in turn increases central expression of TNF-α through the actions of microglia. This increase in TNF-α levels in the brain would then downregulate ApoE expression, and as both ApoE and reelin work through ApoE receptors and may have a synergistic effect on regulating neural plasticity, one could speculate that alterations in ApoE might also indirectly affect reelin binding to ApoE receptors and as a result, reelin functionality would be altered. This process would be reversed upon treatment with TNF-α antagonists.

Another question that remains to be answered is *when* etanercept actually interacts with reelin. In the Brymer et al. (2018) study mentioned above, etanercept was given to the rats semi-weekly during the 21-day period of corticosterone administration. Therefore, the rats received etanercept early on, presumably before any significant stress-related pathology had been created by the corticosterone injections. This experimental design leaves open the question of whether etanercept might have neuroprotective effects rather than antidepressant effects *per se*. It will be important to conduct further studies on this issue, with etanercept given in different animal models of depression at different time periods during and after periods of stress, so that a more complete understanding of the beneficial effects of etanercept can emerge.

## Conclusion

Numerous reports point toward the effectiveness of anti-TNFα drugs for some depression patients. Use of TNF-α antagonists as antidepressants may be particularly important for subpopulations of patients with treatment resistant depression that show high levels of expression of proinflammatory cytokines.

## Author Contributions

All authors contributed to the ideas presented in this review and contributed to editing the manuscript. KB wrote the first draft and developed the figure. LK and HC finalized the manuscript.

## Conflict of Interest Statement

The authors declare that the research was conducted in the absence of any commercial or financial relationships that could be construed as a potential conflict of interest.
